# Designing a Novel Digitally Delivered Antiracism Intervention for Mental Health Clinicians: Exploratory Analysis of Acceptability

**DOI:** 10.2196/52561

**Published:** 2024-04-03

**Authors:** Tashalee Rushell Brown, Habiba Amir, Drew Hirsch, Madeline Owens Jansen

**Affiliations:** 1 Department of Psychiatry and Biobehavioral Sciences University of California, Los Angeles Los Angeles, CA United States

**Keywords:** acceptability, antiracism, clinicians, intervention, interview study, mental health, psychiatry residents, racism, social workers, web-based technology

## Abstract

**Background:**

There is a great need for evidence-based antiracism interventions targeting mental health clinicians to help mitigate mental health disparities in racially and ethnically minoritized groups.

**Objective:**

This study provides an exploratory analysis of mental health clinicians’ perspectives on the acceptability of a web-based antiracism intervention.

**Methods:**

Mental health clinicians were recruited from a single academic medical center through outreach emails. Data were collected through individual 30-minute semistructured remote video interviews with participants, then recorded, transcribed, and analyzed using content analysis.

**Results:**

A total of 12 mental health clinicians completed the study; 10 out of 12 (83%) were female candidates. Over half (7/12, 58%) of the respondents desired more robust antiracism training in mental health care. Regarding the web-based antiracism intervention, (8/12, 67%) enjoyed the digitally delivered demo module, (7/12, 58%) of respondents suggested web-based content would be further enhanced with the addition of in-person or online group components.

**Conclusions:**

Our results suggest a strong need for additional antiracist training for mental health clinicians. Overall, participants responded favorably to novel web-based delivery methods for an antiracism intervention. These findings provide important support for future development and pilot testing of a large-scale digitally enhanced antiracist curriculum targeting mental health clinicians.

## Introduction

Racism or expressions of discrimination are often rooted in implicit bias and stigmatizing beliefs [1]. Currently, racism is known to be a key driver of mental health inequities in ethnoracially minoritized groups who may be victims of discrimination [2]. Such experiences often lead to negative mental health outcomes [2]. Current evidence suggests that Black, Indigenous, and people of color youth and adults experience highly disproportionate rates of delayed diagnosis and treatment of autism spectrum disorder, overdiagnosis of conduct disorder, and underdiagnosis of attention-deficit/hyperactivity disorder [3], overdiagnosis of schizophrenia, overuse of antipsychotics with long-term medical consequences, and the underdiagnosis and treatment of depression [4]. Antiracism is the practice of actively opposing the effects of racism through institutional policies and individual behaviors [5]. Several recent systematic and scoping reviews on antiracism interventions in mental health professions have identified only one relevant randomized pilot study to date [5,6]. Of additional importance is that the authors found significant variability in training methodology, variability of intervention duration, and a lack of sufficient efficacy measurements to evaluate existent antiracism interventions [5,6]. Thus, despite the strong need for evidence-based antiracism interventions targeting racial bias among mental health clinicians, such interventions remain underdeveloped and understudied in the literature. Within this context, evidence-based strategies, such as those based in cognitive-behavioral frameworks, have shown promise in addressing prejudiced thoughts, feelings, and behaviors but have yet to be applied to clinicians [4]. Notably, the delivery of tailored psychoeducational content such as this, has the potential to be greatly enhanced by digital design and delivery methods [3,7]. This is especially poignant given that web-based technologies are known to further augment interventional implementation structures with regard to both flexibility and sustainability [3,7].

Against the backdrop of a profound dearth of evidence-based antiracism interventions targeting mental health professionals, this study aims to explore aspects of the acceptability of a novel digitally delivered intervention of this sort [7]. Grounded in a strongly evidenced implementation science framework and through a dynamic and iterative process of evaluation, we explored facets of intervention acceptability regarding content, delivery, and implementation strategies [8]. Semistructured interviews were designed to elicit additional perceptions and attitudes among mental health professionals regarding gaps and opportunities in their current training on antiracism. Findings have the potential to be incorporated into future modifications of the intervention in order to optimize the feasibility and acceptability of large-scale randomized control pilot trials.

## Methods

### Overview

Participants were residents, fellows, and social workers specializing in mental health care. They were recruited from a single academic medical center in California through a remote method, which included outreach emails. Written, informed consent was obtained from all participants. Participants were compensated through US $50 gift cards and water bottles. Data were collected through individual 30-minute semistructured remote video interviews with participants, which were recorded and transcribed for analysis. Semistructured interview questions were developed based on the clinical experience and literature review conducted by MOJ and TRB (Table 1).

The semistructured interview featured a presentation of a digital demo module of the cognitive behavioral therapy (CBT)–based intervention, which discussed core beliefs that may be harmful in the treatment of patients with mental health conditions. The module features real-world examples, teaches a key concept of intervention, presents examples of self-monitoring, and provides a visual outline of the engagement and reward components. Data were qualitatively analyzed using inductive coding and thematic analysis methods [9] using the Atlas.ti (Scientific Software Development GmbH) software by 2 independent coders (HA and DH). Identified codes and themes were reviewed and consolidated by the leading authors (MOJ and TRB) until consensus was achieved. The number of respondents mentioning each code or theme was reported.

**Table 1:** Semistructured interview questions and probes.

Domain	Questions	Follow-up probes
Sociodemographic information	What is your profession? Where are you in your training?	How would you describe your race and gender?
Definitions and thoughts of antiracism	How would you define antiracism? What terms do you like to use to describe or discuss racism and antiracism?	Are you comfortable talking with coworkers or supervisors about racism and antiracism?
Strengths of the current antiracism training	What are the strengths of your medical training thus far with regard to antiracism?	What training, educational tools or courses have you benefited from in medical school or at the postgraduate level?
Weaknesses of the current antiracism training	What are the weaknesses of your medical training thus far with regard to antiracism?	What additional support, educational tools, or resources would help elevate your clinical skills to provide equitable care to diverse populations?
Feedback on the demo module	How would you describe your experience going through the demo module? What format would you prefer for antiracism training (in person, online, zoom)? Would you have 10-15 minutes to dedicate to this specific type of antiracism learning? Would seeing a report of potential bias in your electronic health record make you more or less likely to complete antiracism training? Why or why not?	What did you find helpful about the demo module? What would make it more helpful? How would you feel about your organization using digital means (online) in the form of self-directed modules to provide antiracism training? Would 10-15 mins, once per week, for 6 weeks seem manageable? A report of potential bias may include: Frequency of biased statements in notes Racial disparities in prescribing patterns

### Ethical Considerations

This study was approved by the University of California, Los Angeles Institutional Review Board (IRB#22-001632-AM-00002).

## Results

A total of 12 mental health clinicians (psychiatry residents, fellows, and social workers) completed the semistructured interviews. The participant characteristics included: female candidates (10/12, 83%), male candidates (2/12, 17%), and Asian (5/12, 42%), Black (2/12, 17%), Hispanic or Latinx (1/12, 8%), Middle Eastern (1/12, 8%), multiracial (1/12, 8%), White (1/12, 8%), and other (1/12, 8%) candidates.

The results of the content and thematic analysis are summarized in Table 2, but major themes are highlighted as follows: the majority of participants (7/12, 58%) desired more robust antiracism training in mental health care. With regard to the demo module, the majority (8/12, 67%) enjoyed the module, (6/12, 50%) found it to be well-organized, and (11/12, 92%) felt the time commitment to be manageable. Many participants particularly enjoyed the CBT-based content (4/12, 33%), especially the daily self-reflection log (4/12, 33%). About 4 participants expressed a preference for an online self-directed structure, and 7/12 (58%) participants suggested that online content could be enhanced with an in-person or group component. Lastly, 4 participants communicated ways to improve participant engagement through the digital modality, including offering incentives, sharing personal experiences, and recording progress.

**Table 2:** Themes and representative quotes from semistructured interviews.

Question	Themes	Quotes
Discussions about race	Comfortable (5 mentions) Somewhat comfortable (2 mentions) Not very comfortable (4 mentions)	I think so … I have to admit that oftentimes in the face of authority figures, it can be challenging…, it can get tiring though, when you’re one of the few faces of color, or if you’re like, the only Black person in the room…
Definitions of racism and antiracism	Active advocacy against racism (11 mentions) Racism as all-encompassing and systemic (5 mentions) Self-awareness of antiracism (4 mentions)	Antiracism, specifically, is a life-long journey, being aware of racial dynamics and disparities and power dynamics, I see it as, like, a modifiable factor.
Previous antiracism training	Beneficial, in-depth discussions and courses at some point (11 mentions) Limitations of training format and practicality (8 mentions) Strong training in residency (7 mentions) Minimal or no antiracism training (6 mentions) Insufficient institutional support (5) Beneficial scenario training (4 mentions)	A lot of it is very theoretical; less of it is practical in the sense of, you know, in a specific situation. I feel like there’s a lot of, like, resident-driven antiracism efforts … justice, equity, diversity, and inclusion groups… Anti-racist work has been performative, ...there was too high a burden on faculty of color…
Antiracism training needs	More robust training and resources (7 mentions) Integration of representation and lived experiences (4 mentions) Accessible language (4 mentions) Integrate translational social sciences in curriculum (3 mentions) Increase cultural competency (2 mentions) Mitigate minority tax (2 mentions)	Just hiring, you know, more faculty of color, I feel that the best ways I’ve learned have been when developing relationships outside of academia bubbles and being with people with lived experience. Having more, like, role-playing kind of activities might be great because for me, it’s like if I’m in a situation where I have to speak up, my mind goes blank.
Digital demo module experience	Enjoyed digital module (8 mentions) Clear and organized web-based structure (6 mentions) Particularly liked CBT-based examples of core beliefs (4 mentions) Particularly liked online daily self-reflection logging (4 mentions)	I really like the module. That’s just like what happens at the hospital. It was clear and I thought the structure was very helpful and consistent while going through the four examples of core beliefs The good clinician one in particular led me to think about how there are so many ways the system rewards not thinking and not challenging biases, and I think it was nice that you provided that example.
Digital demo module time	Feels that 15 minutes/week of web-based intervention content for six weeks is manageable (11 mentions)	Yes, I think we can definitely make that time.
Demo module digital format	Prefers online content with addition of in-person or group setting (7 mentions) Concerns about exclusively online, self-directed formats (5 mentions) Prefers self-directed online-only modules (4 mentions) Open to conducting over a Zoom call (3 mentions)	In-person is generally always the most effective. I think we tend to have short attention spans, and it becomes just an online module you have to do. If you really want people to be an active participant and really engage with it, I don’t know how good self-directed modules are … I’m just like clicking through it.
Digital demo module improvements	Enhance resident participation and engagement in format (4 mentions) Include web-based incentive to track growth (2 mentions)	I think that it might be helpful to allow us space to bring up our own examples, but I know that it takes a lot of vulnerability for us to sit there and reflect. If there’s some sort of incentivization structure for people to check back in or record progress into, like a diary, I think that could be effective.
Potential report of EHR bias	Yes, it would be helpful (11 mentions)	Yeah, it would overall. I think it would be cool, because in the same way that they make us look at how often we are prescribing benzos, why can’t we also be explicit, you know. in terms of antiracism? Yeah, it would make me more wary. It would make me sit down and think.
Across questions	Minority tax (5 mentions)	Everyone has the responsibility to care for, like, a language diverse community. It [shouldn’t] just fall on certain individuals just because of their background. I remember there were some moments in medical school where I felt like there was too high a burden on faculty of color and also students of color.

## Discussion

### Overview

Using semistructured interviews with mental health professionals, our results indicate favorable acceptability of antiracist intervention content and digital delivery methods. The web-based demo module of the antiracism intervention received a high level of positive feedback, with participants finding it relevant, well-structured, and generally effective in teaching CBT principles. For example, participants enjoyed learning how to identify, react to, and consciously correct core beliefs that propagate racism in health care. Regarding acceptability, participants felt the time commitment would be feasible, especially the convenience of being able to access web-based modules for short periods of time over the course of several weeks. Online self-directed training was well-received, with a recommendation for the addition of a group, in-person, or zoom component to solidify and expand upon web-based self-directed learning. Participants also felt that this would improve engagement, especially with opportunities to share their own experiences. Such findings are in line with previous research suggesting that personalization and increased social connectedness facilitated by digital health intervention components can enhance user engagement [10].

In the context of existing literature, there is a need for targeted evidence-based antiracist strategies addressing the unique and specific needs of clinicians operating in any given health care specialty, as the needs of most mental health professionals will differ greatly from those of general health practitioners [11]. Unfortunately, most antiracism interventions to date have focused only on general health professionals, resulting in the existence of far less tailored interventions addressing a specific health care context or specialty. Furthermore, there are limited discussions of methods for enhancing engagement in antiracism training other than mandating antiracism work [11]. Findings from this study fill this critical gap in the literature by investigating needed aspects of antiracist intervention dedicated to specialized mental health care, with the added benefit of using novel digital-based design elements promoting enhanced acceptability and participant engagement.

### Limitations

Limitations include the fact that this study was conducted at a single academic center, which limits its generalizability to other institutions. However, this is a targeted approach to be applied to the study population of mental health professionals. A similar approach can be applied to other health specialty areas, using interviews targeting clinicians of interest. Such methods may further be used to tailor digital antiracism training to other clinical specialties. Another limitation is that the current study focuses on the acceptability of the intervention rather than its efficacy. Lastly, another important limitation lies in the lack of community engagement in the intervention design process, an aspect known to enhance the health equity of digital health interventions [12,13]. Future iterations will therefore aim to involve the systematic incorporation of the voices of community members served by mental health professionals.

### Conclusions

Taken together, these results provide important guidelines for the implementation of a targeted intervention for mental health clinicians. They suggest favorable acceptability regarding the use of CBT principles in antiracism education and delivery in a web-based format. Such synthesized findings and insights from mental health professionals may be used to tailor and guide practical aspects of the further development and piloting of a future large-scale web-based antiracism intervention.

## Abbreviations

CBT: cognitive behavioral therapy

## Data Availability

The data used for this study are available from the corresponding author upon reasonable request.
